# Performance of a predictive algorithm in sensor-augmented pump therapy in the prevention of hypoglycaemia

**DOI:** 10.1186/1687-9856-2015-S1-O36

**Published:** 2015-04-28

**Authors:** Mary Abraham, Martin de Bock, Raymond Davey, Michael O’Grady, Trang Ly, Nirubasini Paramalingam, Barry Keenan, Geoff Ambler, Jane Fairchild, Michele O’Connell, Fergus Cameron, Bruce King, Elizabeth Davis, Timothy Jones

**Affiliations:** 1 Princess Margaret Hospital, Perth, WA, Australia; 2 Telethon Kids Institute, UWA, Perth, WA, Australia; 3 Medtronic Minimed, Northridge, California, USA; 4 The Children's Hospital at Westmead, Sydney, NSW, Australia; 5 Women and Children’s Hospital, Adelaide, SA, Australia; 6 Royal Children’s Hospital, Melbourne, VIC, Australia; 7 John Hunter Children's Hospital, Newcastle, NSW, Australia

## 

The Predictive Low Glucose Management (PLGM) system consists of a Medtronic Veo pump, Enlite sensor, MiniLink REAL-Time transmitter, Bluetooth-RF translator and a predictive algorithm operating from a Blackberry smartphone. The system suspended insulin delivery when the pre-set hypoglycaemic threshold of 4.4mmol/L was predicted to be reached in 30 minutes.

The aim of this study was to determine the plasma glucose profile with the PLGM system when hypoglycaemia was induced by (a) moderate-intensity exercise, (b) subcutaneous insulin bolus and (c) increasing the overnight basal infusion rate in individuals with type 1 diabetes. The primary outcome was the plasma glucose nadir following each hypoglycaemic stimulus with and without PLGM.

Participants performed 30-60 minutes of moderate-intensity exercise or were administered a subcutaneous insulin bolus following a glucose stabilisation period on basal continuous subcutaneous insulin infusion. In participants studied with increased overnight basal rates, hypoglycaemia was induced by increasing basal rates by 180%. They were randomised and studied on 2 separate days; with PLGM off and with PLGM on. On both days, participants were observed until plasma glucose dropped to 2.8mmol/L or were symptomatic.

## Results

A) PLGM suspended basal insulin in 9/13 participants who performed moderate-intensity exercise resulting in a higher glucose nadir in 6/9 participants (66%).The mean±SD glucose nadir with PLGM off and on was 3.38±0.44mmol/L and 3.5±0.65mmol/L respectively. (p=0.27)

B) PLGM suspended basal insulin in 27 participants in whom SC insulin bolus was administered resulting in a higher glucose nadir in 20 participants (74%). The mean±SD glucose nadir with PLGM off and on was 3.33±0.47mmol/L and 3.71±0.5mmol/L respectively. (p=0.003)

C) PLGM suspended basal insulin in 6/6 participants in whom overnight basal rates were increased resulting in a higher glucose nadir in 5/6 participants (83%). The mean±SD glucose nadir with PLGM off and on was 2.7±0.29mmol/L and 3.9±1.11mmol/L respectively. (p=0.05)

Sensor-augmented pump therapy with the PLGM system is likely to be effective in reducing the risk of hypoglycaemia. The system appears to be more effective when hypoglycaemia is induced by insulin bolus and increased overnight basal rates than with exercise.

